# Intelligent leak monitoring of oil pipeline based on distributed temperature and vibration fiber signals

**DOI:** 10.3389/fdata.2025.1667284

**Published:** 2025-11-20

**Authors:** Xiaobin Liang, Yonghong Deng, Yibin Wang, Hongtao Li, Weifeng Ma, Ke Wang, Junjie Ren, Ruijiao Ma, Shuai Zhang, Jiawei Liu, Wei Wu

**Affiliations:** 1 Institute of Safety Assessment and Integrity, State Key Laboratory of Oil and Gas Equipment, CNPC Tubular Goods Research Institute, Xi'an, China; 2 Engineering Technology Research Center, Hancheng Gas Production Management Area of PetroChina Coalbed Methane Co., Ltd., Hancheng, China; 3 School of Chemical Engineering, Northwest University, Xi'an, China

**Keywords:** deep learning, safety pre-warning, distributed fiber optic sensing system, leakage monitoring, oil pipeline

## Abstract

Due to long-term usage, natural disasters and human factors, pipeline leaks or ruptures may occur, resulting in serious consequences. Therefore, it is of great significance to monitor and conduct real-time detection of pipeline leaks. Currently, the mainstream methods for pipeline leak monitoring mostly rely on a single signal, which have significant limitations such as single temperature being susceptible to environmental temperature interference leading to misjudgment, and single vibration signal being affected by pipeline operation noise. Based on this phenomenon, this research has built a distributed optical fiber system as an experimental platform for temperature and vibration monitoring, obtaining 3,530 sets of real-time synchronized spatial-temporal temperature and vibration signals. A dual-parameter fusion residual neural network structure has been constructed, which can extract characteristic signals from the original spatial-temporal temperature and vibration signals obtained from the above monitoring system, thereby achieving a classification accuracy of 92.16% for pipeline leak status and a leakage location accuracy of 1 m. This solves the problem of insufficient feature extraction and weak anti-interference ability in single signal monitoring. By fusing the original temperature and vibration signals, more leakage features can be extracted. Therefore, compared with single signal monitoring, this study has improved the accuracy of leakage identification and location, bridging the gap of misjudgment caused by single signal interference, and providing a basis for pipeline leakage monitoring and real-time warning in the oil industry.

## 1 Introduction

With the increasing global demand for energy, pipeline as an important channel of energy transmission, its safe and reliable operation is of great significance to ensure energy supply and social stability. However, due to various reasons such as long-term use, natural disasters and human factors, various failure problems will occur in pipelines. Pipeline leaks or ruptures pose significant hazards, encompassing environmental pollution, substantial economic losses, and even human casualties. Consequently, the implementation of pipeline monitoring and early warning systems is particularly crucial for enhancing operational safety and ensuring energy supply.

At present, the common technology of pipeline leakage monitoring can be categorized as hardware detection and software monitoring according to the main implementation mode (Wu et al., 2023). Hardware detection includes acoustic emission detection, soil detection, ultrasonic detection, cable detection, and distributed optical fiber detection method (Mostafapour and Davoudi, 2013; Leinov et al., 2016; Datta and Sarkar, 2016; Zuo et al., 2020). Software monitoring includes mass (or volume) balance method, pressure point analysis method, pressure gradient method, statistical method, and real-time transient modeling method (Mujtaba et al., 2020; Fukushima et al., 2000; Lu et al., 2020; Abbaspour and Chapman, 2008; Wang et al., 2019). Most of the traditional hardware methods need manual participation, low efficiency, high operating cost, small coverage, easy to be disturbed by human factors, and can't be continuously monitored. As a novel pipeline detection technology proposed in recent years, the distributed optical fiber detection method can achieve uninterrupted parameter acquisition along the optical fiber path. Optical fibers can not only achieve simultaneous sensing and transmission but also possess an extended detection range, high measurement precision, strong corrosion resistance, and immunity to electromagnetic interference. Statistical method and real-time transient modeling method are the most widely used methods with the highest accuracy among software monitoring methods. However, real-time transient modeling needs to collect various detailed parameters of the monitored pipeline, and modeling can be carried out based on these data. The detail and accuracy of parameters will directly affect the accuracy of the model, so it is crucial to collect multi-dimensional parameters from the pipeline being inspected (Arifin et al., 2018; Oseni et al., 2023). The statistical method based on machine learning can analyze the collected pipeline sensor data by statistical method, and train the data by machine learning algorithm to obtain the standard mode of normal operation of the pipeline and identify the abnormal mode when pipeline leakage occurs.

At present, various machine learning models are often used to provide standard patterns, so that they have better accuracy and adaptability. In 2020, Ya et al. (2019); Zhang et al. (2021) used a distributed optical fiber temperature system to conduct pipeline leakage detection and localization experiments. For determining the pipeline's leakage condition, the correlation coefficient method and absolute distance method are used to cluster the temperature detection signal, and the selective average threshold of valid leakage point is used. Liu et al. (2023) proposed a multi-dimensional spatial data fusion algorithm based on the acquisition of time-air pipeline leakage signals by DVS (distributed optical fiber vibration sensing technology) system. The average value of the obtained non-leakage fusion signals was used as the alarm threshold to improve the leakage alarm rate and realize multi-point leakage alarm.

However, in practical applications, the temperature and vibration signal fluctuate when the pipeline leaks, so identifying the leak by setting a fixed threshold can cause false positives and false negatives, especially for small leakage problems. With the rise of machine learning, neural networks can perform statistical analysis on signals and learn the features of global networks. They are gradually used in the identification and classification of distributed fiber signals, and good results have been achieved. Abufana et al. (2020) denoised and filtered distributed optical fiber acoustic signals through wavelet denoising and difference time domain method, used variational mode decomposition to extract features such as variance, skewness, and kurtosis of signals and finally classified signals based on linear SVM (support vector machine). Bao et al. (2020) employed a combined endpoint detection and variational mode decomposition approach for extracting time-frequency characteristics from fiber vibration signals, and then three different intrusion signals were identified by SVM model with a recognition accuracy of 98%. Wang et al. (2021) integrated the distributed vibration and temperature system, extracted 6 temperature and 5 vibration characteristic values, and identified the operating state of the pipeline through the random forest model.

However, artificial feature extraction usually increases the computing resources of the recognition algorithm, resulting in low processing efficiency and poor real-time performance. Therefore, more and more studies consider deep learning models to integrate feature extraction and classification recognition to improve the accuracy and real-time performance of the models. In 2023, Li et al. (2023) adopted the CNN-LSTM structure to analyze the temporal and spatial features of the signal, and also used the double cubic reduction to simplify the network structure, and realized the vibration event recognition of the buried distributed fiber optic sensing system. Observations from numerous recent studies indicate that deep learning models typically outperform machine learning models in both recognition accuracy and computational speed, and in terms of model computing efficiency, deep learning also has a faster recognition speed (Lyu et al., 2020; Xie et al., 2022; Zhu et al., 2023). Shi et al. (2020) acquired spatio-temporal signals based on the DVS system, converted the signals into grayscale graphs, extracted signal features, and classified them by 2DCNN-SVM model, with an accuracy rate of 94.17%. Peng et al. (2020) used a DAS (distributed optical fiber acoustic sensing technology) system to monitor acoustic signals from pipelines, and compared the results of shallow un-convolutional neural networks with the CNN smodel. The results showed that the CNN model had higher event type recognition accuracy. Yang et al. (2022) proposed an integrated 1DCNN-VAPSO-SVM model utilizing pipeline acoustic signals for leakage detection. And the parameter combination in SVM was optimized by adopting an amplitude-based parameter adjustment strategy, effectively improve the accuracy of the model. Zhang et al. (2023) proposed AM-LSTM model to identify monitoring signals of time series and realize real-time monitoring and location of pipeline leakage.

Comprehensively considering the research and application status of deep learning models in the field of distributed fiber signal recognition, it can be seen that simultaneous feature extraction and classification recognition of signals through deep learning models is a feasible method to improve the intelligence of signal recognition. In addition, considering the space-time dimension of fiber signals in the model also can effectively improve the signal characterization ability. However, a deep learning model that is too complex will also reduce real-time signal recognition. Therefore, how to optimize the accuracy-real-time performance trade-off in deep learning models is a problem that must be considered to realize intelligent monitoring of pipeline leakage.

In addition, most studies mainly focus on the vibration, strain, temperature and acoustic signals of distributed optical fibers, and few studies mix a variety of signals together to identify pipeline leakage status. In fact, due to the high temperature and pressure of oil in the oil pipeline, the leakage of the pipeline will produce vibration and accompanied by the phenomenon of temperature rise. But when the leakage is small, the minute temperature or vibration variations induced by leakage, making their timely detection challenging using individual signals. In order to solve this problem, the temperature and vibration signals can be considered simultaneously, so that the impact of environmental interference can be reduced, and more information can be extracted to improve the accuracy of leakage identification (Wang et al., 2021).

Taking the above reasons into consideration, this study proposes an intelligent oil pipeline leakage monitoring method based on spatio-temporal signals of the distributed optical fiber vibration and temperature detection systems. The deep residual network is used to characterize the features and states of the vibration and temperature signals of distributed fiber with spatio-temporal dimensions to obtain the pipeline leakage status. This model can quickly process the collected original data, realize intelligent real-time monitoring of pipeline leakage, and accurately locate the leakage point. The main research questions and objectives of this study are as follows.

Research Questions (RQs):

RQ1: How to build an experimental system that can stably obtain high-quality spatial-temporal synchronized temperature-vibration signals?
RQ2: How to enhance the feature extraction ability and anti-interference performance of pipeline leakage and solve the inherent defects of single-signal monitoring?
RQ3: What level of accuracy and localization precision can be achieved with the proposed dual-parameter fusion method?

Research Objectives (ROs):

RO1: A self-developed distributed optical fiber temperature and vibration monitoring experimental platform has been built. A total of 3,530 sets of real-time synchronized spatial and temporal temperature and vibration signals have been successfully collected, which ensures that the data cover the signal characteristics of the pipeline under both normal and different leakage conditions and meet the data quality and quantity requirements of the leakage monitoring model.
RO2: The model is used to extract features from the original vibration signal and temperature signal, respectively. Through decision-level fusion, the advantages of larger amplitude changes in DVS during leakage and a 1-meter accuracy of DTS are combined, thereby breaking the limitations of a single signal.
RO3: A dual-parameter fusion residual neural network structure was constructed, which solved the problems of gradient vanishing and slow convergence during the training process when traditional CNNs learned more complex deep feature models. Eventually, a classification accuracy of 92.16% for pipeline leakage and a leakage location accuracy of 1 m were achieved.

## 2 Preliminaries

In this section, the fundamental theories, concepts and advantages of distributed optical fiber system, convolutional neural network (CNN), and residual network (ResNet) are briefly outlined.

### 2.1 Distributed optical fiber system

A distributed optical fiber sensing technique employs a novel sensing technology that directly uses the monitoring optical cables laid in the same trench as pipelines as sensors. It fully exploits the characteristics of continuous spatial distribution in fiber, forming the integration of “transmission” and “sensing,” and enabling the acquisition of physical parameter information at any point along the fiber. It can be used for applications in the fields of various industries including petroleum, petrochemicals, electric power, transportation, and bridge construction. Compared with traditional detection technologies, this technique has the advantages of long measurement distance, continuous distributed measurement, accurate positioning, simple installation, high safety and strong scalability.

**Figure 1:**
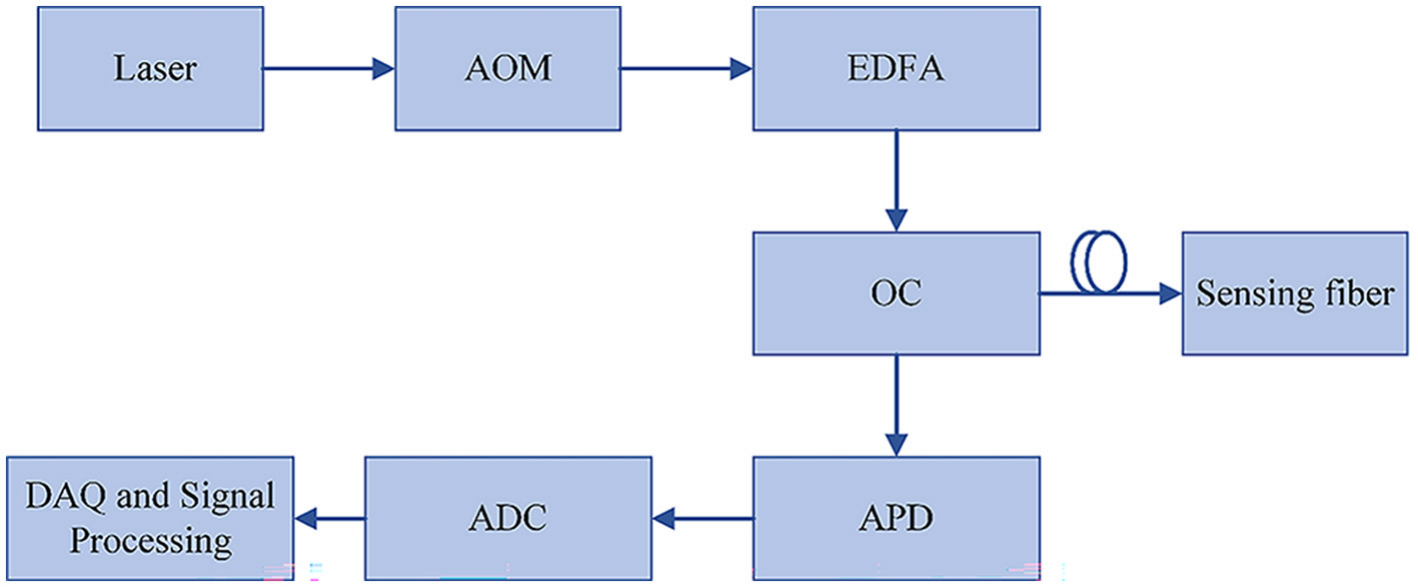
Typical structure of optical fiber sensing system.

There are three types of scattered light that occur when light is transmitted within the fiber, which are Rayleigh scattering, Brillouin scattering, and Raman scattering. Based on these scattering types, the optical fiber can realize the detection of vibration, temperature and other signals. Figure 1 shows the typical architecture of a common distributed optical fiber sensing system.

The DVS system is founded on the principles of Rayleigh scattering and ϕ-OTDR (phase-sensitive optical time domain reflectometry). The optical modulator modulates the continuous light emitted by the narrow linewidth laser with strong coherence into pulsed light in this system. The pulsed light continuously generates coherent back-Rayleigh scattering light modulated by external vibration signals when propagating in the fiber. When the fiber is in a stable state, the variation law of Rayleigh scattering intensity is basically unchanged. But when a certain section of the fiber vibrates, the refractive index and length of the corresponding position will change, leading to a change in the phase of the scattered light from the point of scattering. The change of the relative phase relation of the scattered light will lead to the fluctuation of the intensity of the back-Rayleigh scattered light under the action of light interference, and the change frequency is highly related to the external vibration frequency. Therefore, the vibration information can be obtained by calculating the intensity change of the scattered light, and the optical power can be obtained by the photodetector to detect sensing signals of DVS.

The DTS system is based on the Raman scattering and ϕ-OTDR principles. The properties of Raman scattering are used to measure ambient temperature. After Raman scattering, the incident light can produce two different frequencies of light. The frequency of Stokes light is lower than incident light and its intensity is independent of temperature, while anti-Stokes light possesses a greater frequency compared to incident light and its intensity is affected by temperature. Therefore, the temperature of the region can be obtained by measuring the intensity proportion of Stokes light vs. anti-Stokes light. By using OTDR technology, different temperature change points can be located according to the time difference between the incident light and the backward Raman scattering light and the transmission rate of light in the fiber.

### 2.2 Convolutional neural networks

The Artificial Neural Network (ANN) serves as a crucial cornerstone within artificial intelligence and machine learning, mirroring the functionality of biological neural networks. To simulate the role of neurons and synapses in biological neural networks, ANN is composed of a large number of nodes, and these nodes transfer information through connections. Each connection has a weight that indicates the strength of the signal transmission. The main goal of ANN is to solve complex problems through learning and training, such as image recognition, object detection, pattern recognition, text classification and so on.

The Convolutional Neural Network (CNN), a prominent deep learning architecture, has emerged as the predominant artificial neural network for leakage monitoring applications due to its hierarchical feature extraction capability through successive convolutional, activation, pooling, and fully-connected layers (Le Cun et al., 1989; Gu et al., 2018).

The convolutional layer employs trainable kernels that convolve with input data for hierarchical feature extraction. The filters slide over the input, calculating the dot product between their weights and local areas of the input, resulting in a feature map containing different aspects of the data. The activation function typically follows each convolutional operation. By using the activation function, nonlinear operations can be introduced into the network model, so as to learn more complex features. Pooling layers perform dimensionality reduction via down-sampling while preserving salient features, thereby enhancing computational efficiency, preventing overfitting, and improving model robustness and generalization. The network architecture terminates with fully connected layers that leverage the extracted feature representations to execute final classification or regression operations.

Because CNN is able to automatically extract and learn features from inputs, this makes it well suited for applications that require high accuracy and robustness, including pipeline leak detection, where the presence or absence of leaks can be discovered by extracting features and patterns in sensor data.

### 2.3 Residual network

While conventional deep convolutional neural networks theoretically achieve enhanced feature extraction through sequential stacking of convolutional and activation layers, practical implementations often encounter optimization challenges including gradient vanishing and explosion phenomena as network depth increases. As the networks advance to a fairly deep layers, model training becomes more difficult. The phenomenon of gradient disappearance means that during the backpropagation of gradient information, the gradient gradually becomes smaller, resulting in the weight update of the earlier layer becomes very slow, which may cause the network convergence speed to slow down, and even the training failure. To solve these problems, researchers have proposed the residual network (ResNet). Based on the original CNN, the concepts of residual block and skip connection are introduced, in which residual block is the basic building block and core architecture of ResNet. K. He believes that the core idea of the residual network is to directly transfer information from the previous layer to the subsequent layer through skip connection (He et al., 2016). Therefore, the input is added directly to the output through the proposed skip connection, so that the gradient can be propagated more easily through the network. The residual block consists of the mapping part and the residual part, and the mathematical expression is as follows:


$$\begin{array}{l} x_{l+1}=h\left(x_{l}\right)+F\left(x_{l},W_{l}\right) \end{array}$$ (1)

Where, *h*(*x*_*l*_) represents the mapping part, which is used to raise or decrease dimension, usually in the form of direct mapping or 1 × 1 convolution operation, and *F*(*x*_*l*_, *W*_*l*_) represents the residual part.

**Figure 2:**
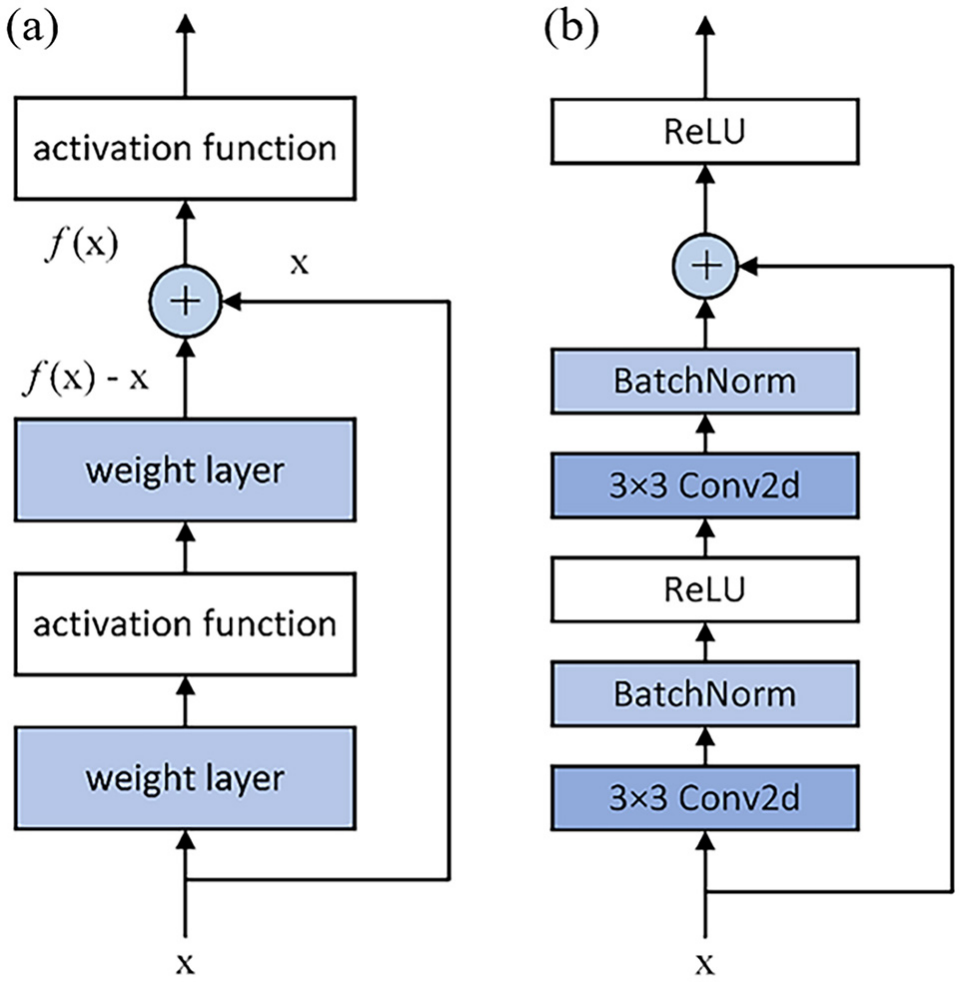
Structure of residual block. **(a)** Structure diagram of residual block; **(b)** Specific structure of residual block.

The structure of the residuals block is shown in Figure 2a. After the introduction of jump joins, the residual function can be expressed as *F*(*x*) = *f*(*x*)−*x*, and when *F*(*x*) = 0, an identity map *f*(*x*) = *x* is formed. When the neural network layer is an identity mapping, the residual function to be learned is 0, which reduces the difficulty of model learning, and the identity mapping also alleviates the problem of model degradation.

The residual architecture facilitates direct feature propagation from input to weight layer outputs through skip connections, effectively mitigating signal attenuation in deep networks. This structural design not only alleviates gradient vanishing during backpropagation—thereby preventing model degradation—but also reduces parametric complexity while enabling effective training of deep neural architectures. As illustrated in Figure 2b, conventional residual blocks employ this mechanism to achieve substantial performance improvements through optimized information flow and gradient propagation pathways.

In the actual network construction, by stacking multiple residual blocks, the network can train the deep structure more easily, thus improving the performance of the model.

## 3 Proposed method

**Figure 3:**
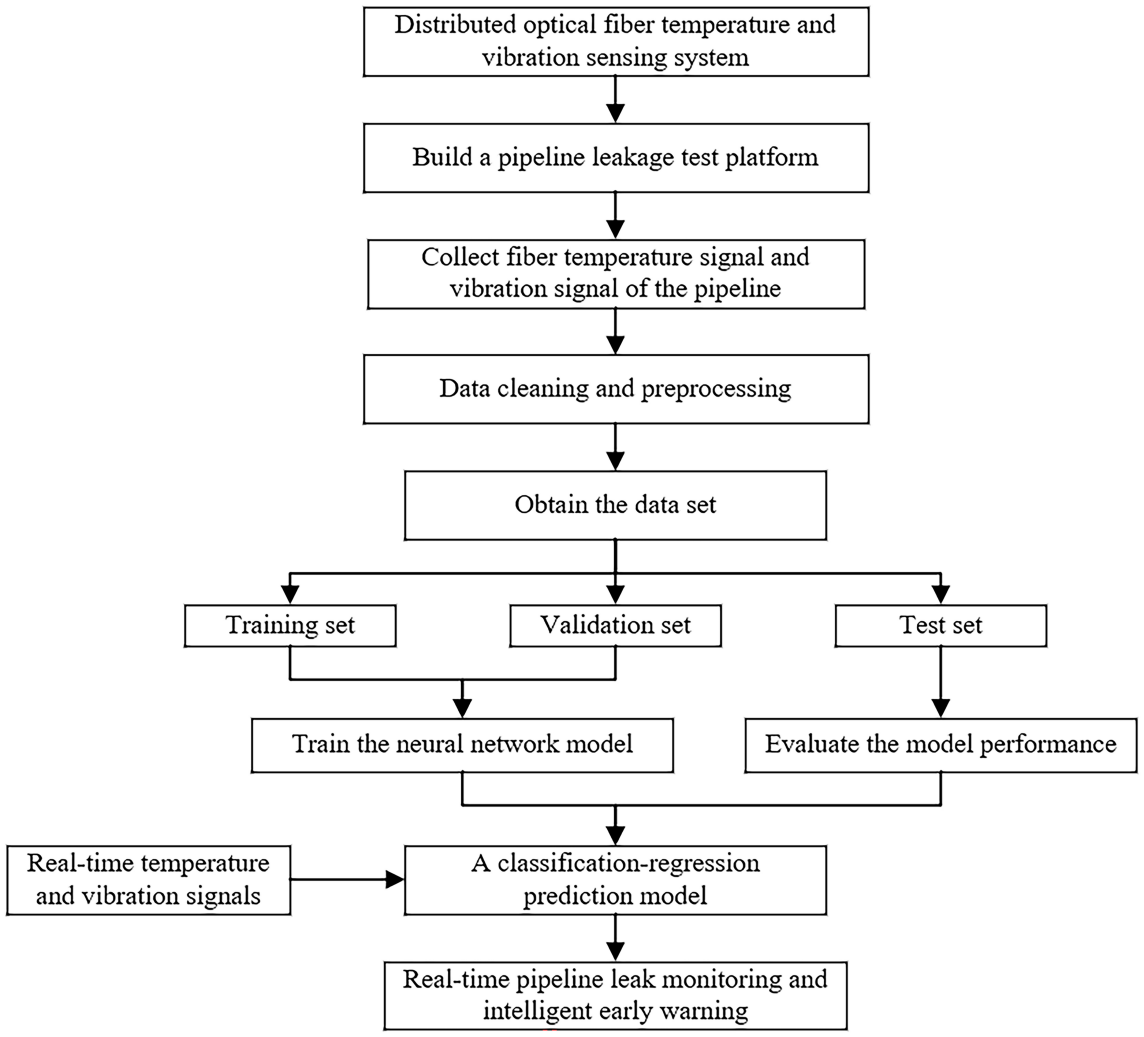
The research frame diagram.

The proposed methodology's architectural overview is illustrated in Figure 3.

With the rapid development of deep learning, CNN, RNN, LSTM, Transformer, and other models have emerged one after another. Each model excels at specific tasks with its unique structure and design principles, however, choosing the right neural network model remains a challenge when faced with different application scenarios. The selection of neural network architecture should be determined based on task requirements, input-output data characteristics, and empirical training performance. The appropriate model structure and parameters often have good accuracy and generalization. The input data of this study are the vibration signal and temperature signal of the pipeline with temporal and spatial characteristics. The integrated analysis of these dual-signal characteristics enables simultaneous pipeline leakage detection and localization, constituting a dual-task learning framework classification and regression. Therefore, we may need to choose a more in-depth and complex model. A residual network is proposed to establish a pipeline leakage monitoring model.

### 3.1 Experimental platform construction (Findings for RQ1)

To ensure model generalization and prevent overfitting, substantial training datasets are essential for machine learning algorithms. But the fiber optic system in the actual pipeline has few signals in this study. Thus, it is necessary to build an experiment platform with DTS and DVS systems on the pipeline to simulate the operating state (normal operation and leakage state) of the actual pipeline. The DTS and DVS systems are used to collect sufficient temperature and vibration signals of the pipeline under normal operation and leakage state, and fuse these two types of signals in the machine learning model, carry out feature extraction, and complete the classification and regression tasks to obtain the pipeline operation state. The experimental test bench enables controlled leakage simulations to generate extensive datasets, ensuring sufficient training samples for neural network models while minimizing statistical uncertainties.

**Figure 4:**
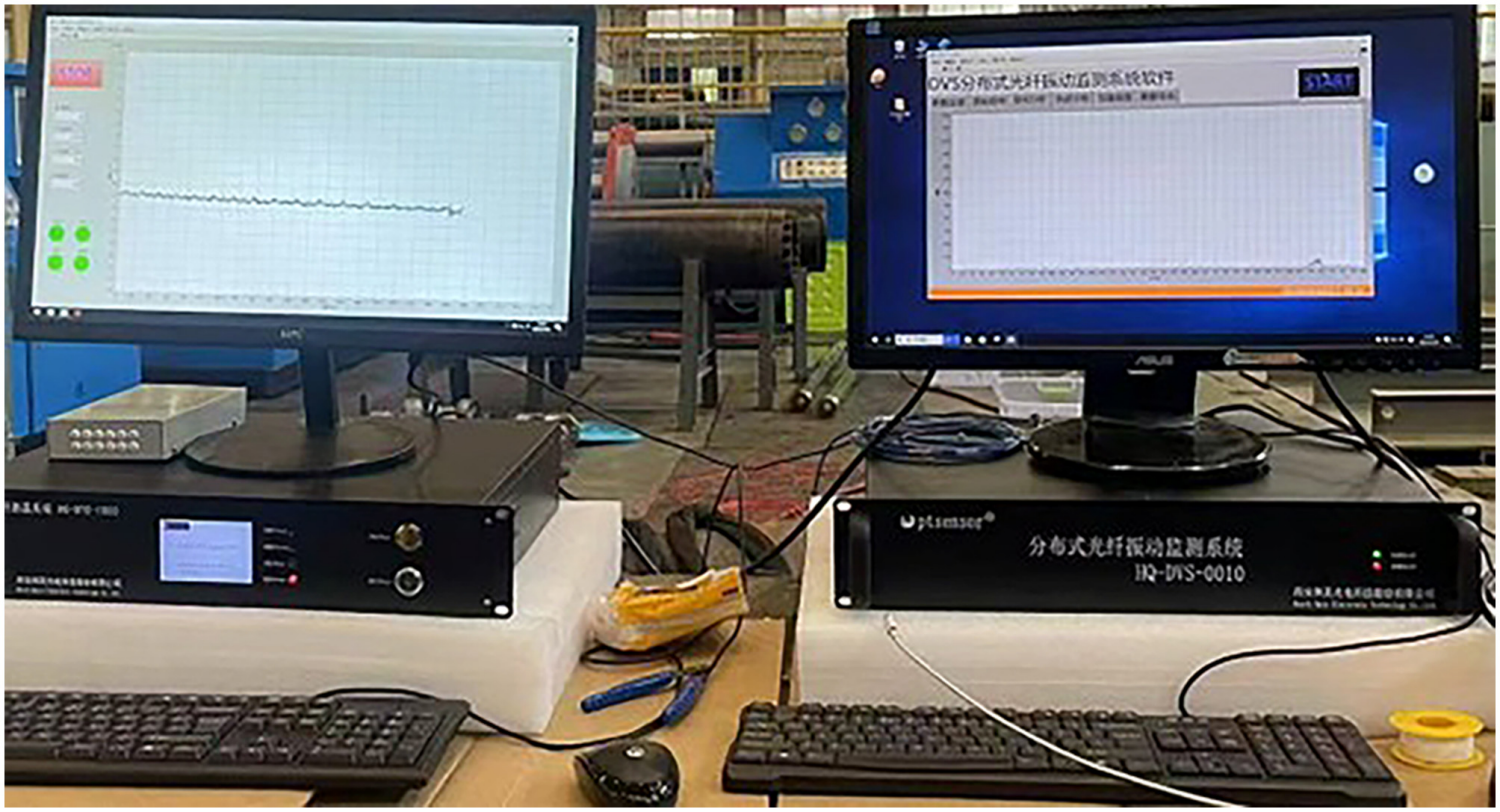
Physical demonstration of distributed optical fiber system.

The DTS and DVS used in this experimental platform are based on an Advantech as their mainboards. The finished product is manufactured by Herch Opto Electronic Technology Co., Ltd. This research employs dual distributed fiber optic sensing systems: a Raman scattering-based temperature monitoring system and a Rayleigh scattering-based vibration detection system, with the experimental setup illustrated in Figure 4.

**Figure 5:**
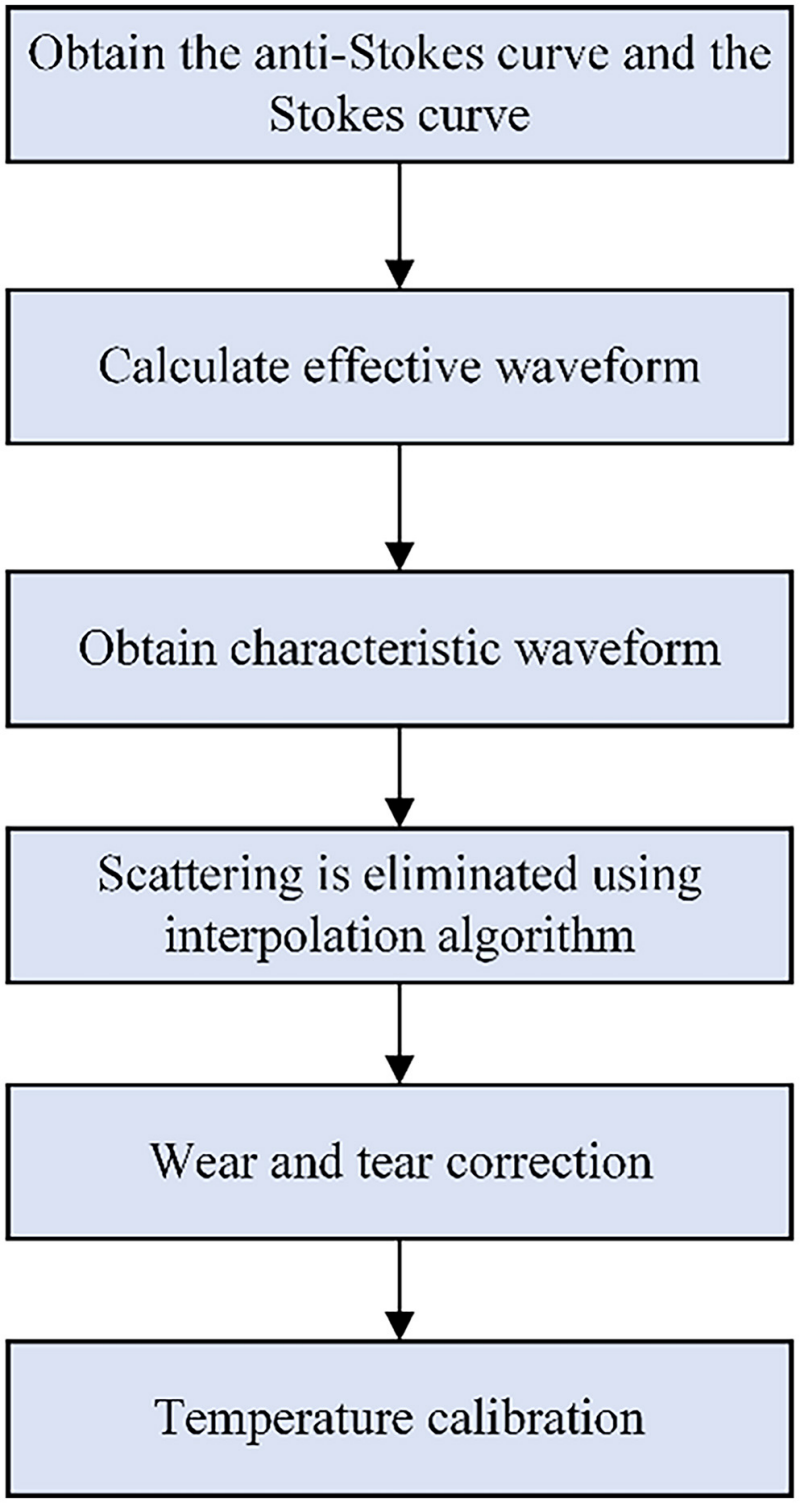
Distributed optical fiber temperature calibration algorithm.

The DTS system adopts the temperature calibration algorithm, the steps of which are shown in Figure 5. This algorithm can reduce the influence of light scattering and transmission loss, thereby improving the accuracy of this DTS system. The technical specifications of DVS and DTS are respectively shown in Tables 1, 2.

**Table 1:** Technical specifications of DVS system.

**Parameters**	**Specification**
Model number	HQ-DVS-0010
Working wavelength	1,550 nm
Monitoring distance	1–10 km
Spatial resolution	±5 m
Frequency range	0.1–2 kHz
Sampling frequency	400 times/min
Response time	≤ 2 s

**Table 2:** Technical specifications of DTS system.

**Parameters**	**Specification**
Model number	HQ-DTS-0010
Monitoring distance	0–30 km
Temperature measurement accuracy	±1 °C
Temperature measurement resolution	0.1 °C
Spatial resolution	1 m
Sampling frequency	27 times/min
Response time	2 s

**Figure 6:**
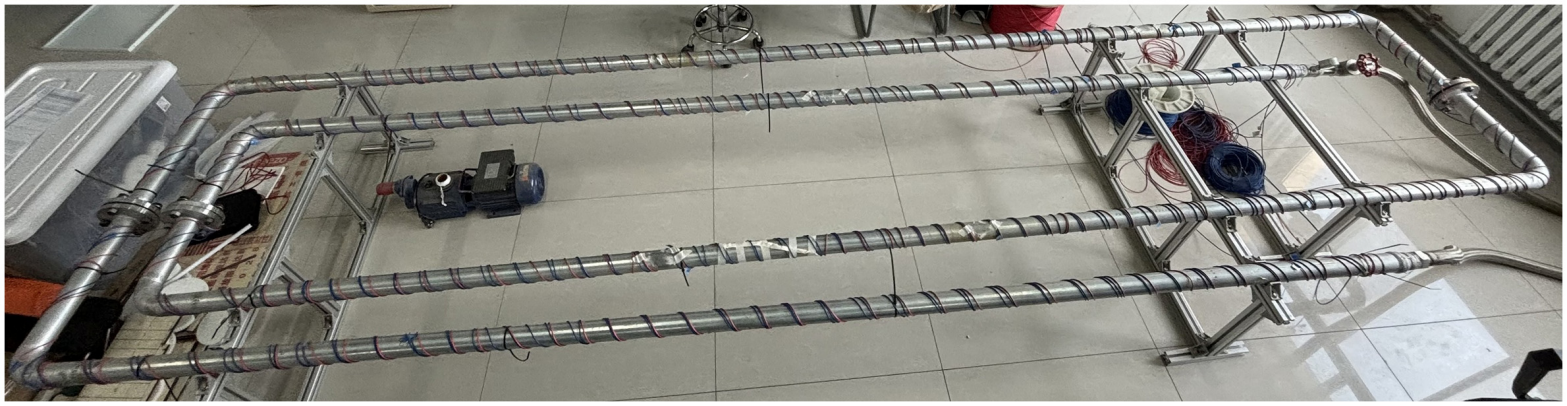
Physical diagram of the experimental platform for monitoring pipeline leakage using distributed optical fiber sensing system.

The experimental setup consists of a 20-meter-long steel pipeline with 60 mm diameter (Figure 6), featuring multiple artificially created leakage orifices of 2 mm and 3 mm diameters sealed by threaded fasteners along the pipe wall at varying intervals. By changing the diameter and spacing of leakage holes, the influence of pipeline leakage on the monitoring results can be reduced, and the resolution of leakage location of the model can be easily determined. The distributed temperature fiber and the vibration fiber are fixed to the pipeline through cable ties. A water pump is connected to the inlet of the pipeline, and a valve and pressure gauge are installed at the outlet. The water flow pressure in the pipe can be adjusted through the valve to the maximum of 1MPa. During the experiment, the non-leakage state of the pipeline was simulated by sealing the nut at the leak hole, and the leakage of different diameters could be simulated by unscrewing the nut of the leak hole. In addition, in order to enable the model to monitor multiple leaks, a series of experiments with two sets of leak holes were also conducted. Taking into account the temperature variation, the duration of each set of experiments was 120 s.

Although the sampling frequency of distributed fiber is 1 KHz, due to the need for filtering and preprocessing of optical fiber signals, as well as temperature calibration of optical fiber temperature signals, these operations lead to a decrease in the signal output speed of the distributed optical fiber system. Finally, during the experiment, the time length of vibration signal obtained from the distributed fiber vibration system for each sample is 800, and the time length of temperature signal read from the distributed fiber temperature system is 54.

**Figure 7:**
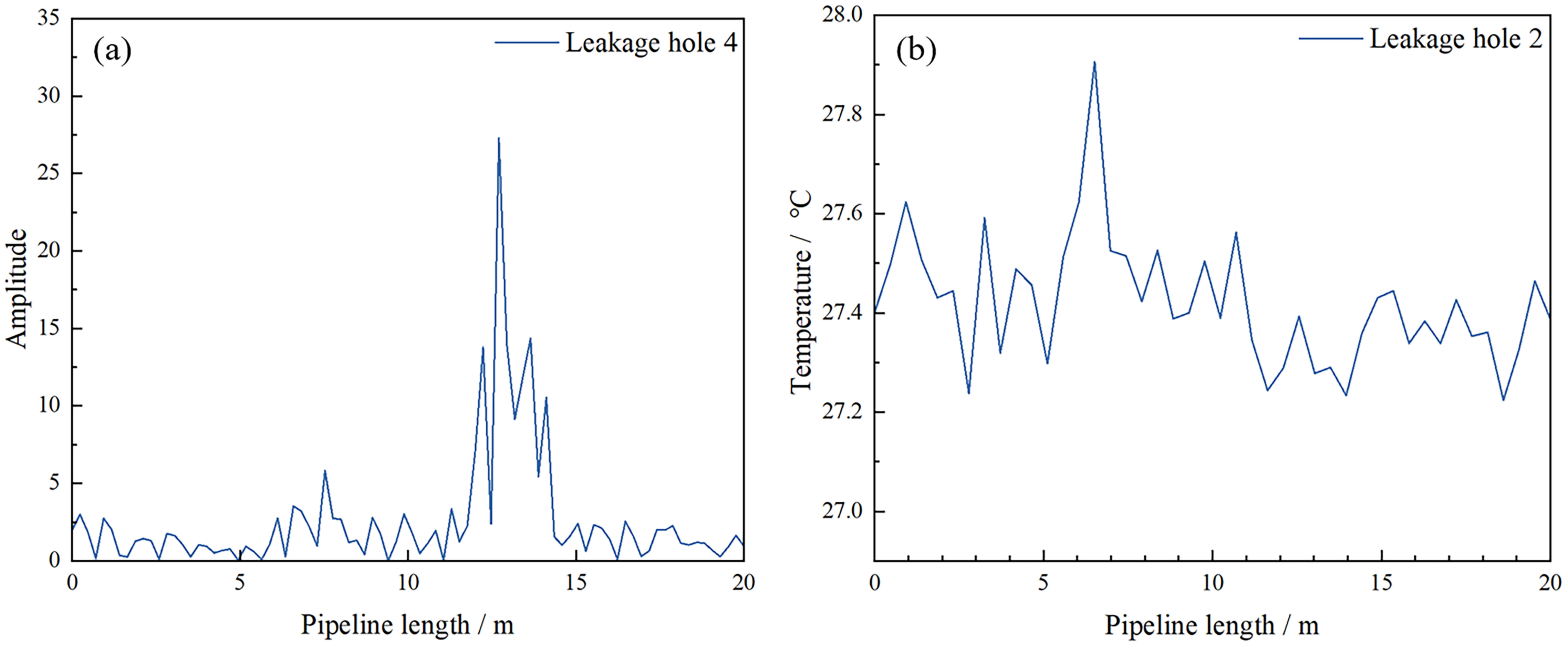
Measurement results of vibration signals **(a)** and temperature signals **(b)** of the experimental pipeline.

**Figure 8:**
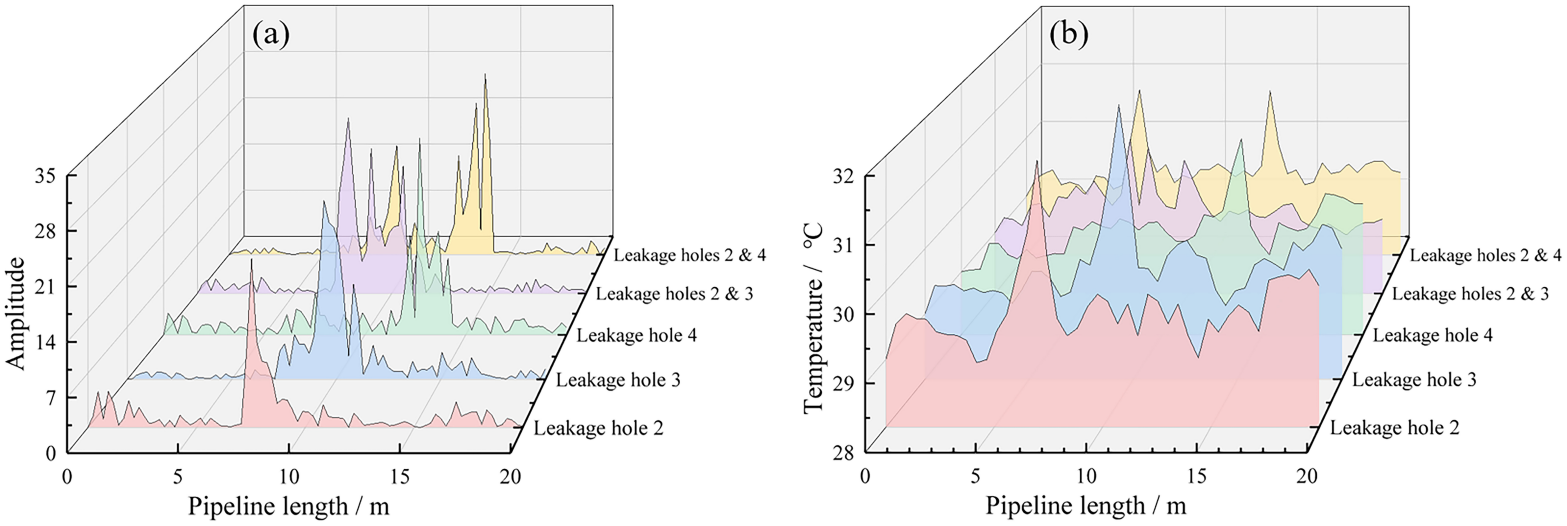
Vibration **(a)** and temperature **(b)** signal graphs of different leakage holes.

Figure 7 shows the temperature signal and vibration signal of a sampling time point read through the distributed fiber system. The results show simultaneous temperature increase and vibration amplification at the leak location. However, since leakage is a long-time process, the introduction of time dimension enables the model to extract more characteristic quantities of leakage signals and judge the pipeline state more accurately. Interference events caused by changes in temperature and vibration signals caused by human and environmental factors can be better identified from the perspective of data samples. Figure 8 presents the distribution characteristics of different leakage holes along the pipeline length through two dimensions of amplitude and temperature. Different color layers correspond to different combinations of leakage holes (such as single-hole leakage, multiple-hole simultaneous leakage), and can be used to analyze the influence patterns of leakage on the vibration and temperature field of the pipeline. In the vibration amplitude distribution under different leakage hole conditions, the higher the peak value, the stronger the vibration caused by the leakage at that position. The temperature change triggered by the leakage will lead to an abnormal increase in local temperature. For multi-pore leakage, the range and intensity of amplitude and temperature enhancement can be increased.

As RO1 stated, this study collected a total of 3,530 sets of usable signal samples. Table 3 lists the number of signal samples and related parameters. This study collected 1,813 sets of normal operating data and 1,717 sets of leakage operation data. Under the leakage condition, two different diameters of leakage holes were set up, and each leakage hole was equipped with a mechanical nut. The mechanical nut could adjust the size of the leakage volume, and different leakage situations ranging from slight leakage to heavy leakage were collected. This enables a more comprehensive assessment of the model's identification and classification capabilities. Additionally, datasets of single-hole leakage and simultaneous multi-hole leakage were also collected. By locating the leakage holes, the positioning accuracy of the model can be further tested.

**Table 3:** The relevant parameters of the collected signal samples.

**Signal samples**	**Number**
Normal	1,813
Leakage	1,717
Time	120 s
Length of vibration signal	800
Length of temperature signal	54

### 3.2 Construction of neural network model

The experimental dataset was constructed by extracting all 3,530 synchronized temperature and vibration signal pairs from the distributed optical fiber system's TDMS output files, which were subsequently normalized to consistent dimensions and integrated into a unified JSON format suitable for deep neural network training. Following standard machine learning protocols, the compiled dataset underwent randomized partitioning into three distinct subsets (training, validation, and test sets) as detailed in Table 4, where the training set facilitates model parameter learning, the validation set enables hyperparameter optimization and interim performance assessment, while the held-out test set provides an unbiased evaluation of the model's generalization capability on previously unseen data. This systematic data preparation and partitioning approach ensures rigorous model development and reliable performance estimation.

**Table 4:** Distribution of the dataset.

**Data set**	**Number**
Training set	1,994
Validation set	768
Test set	768
Total	3,530

In this study, the model construction is based on the Pytorch platform. Due to the temporal and spatial characteristics of fiber optic data, the ResNet model architecture consisting of two-dimensional convolution layer is used to extract the features of the data and complete the task of pipeline status identification and location.

**Figure 9:**
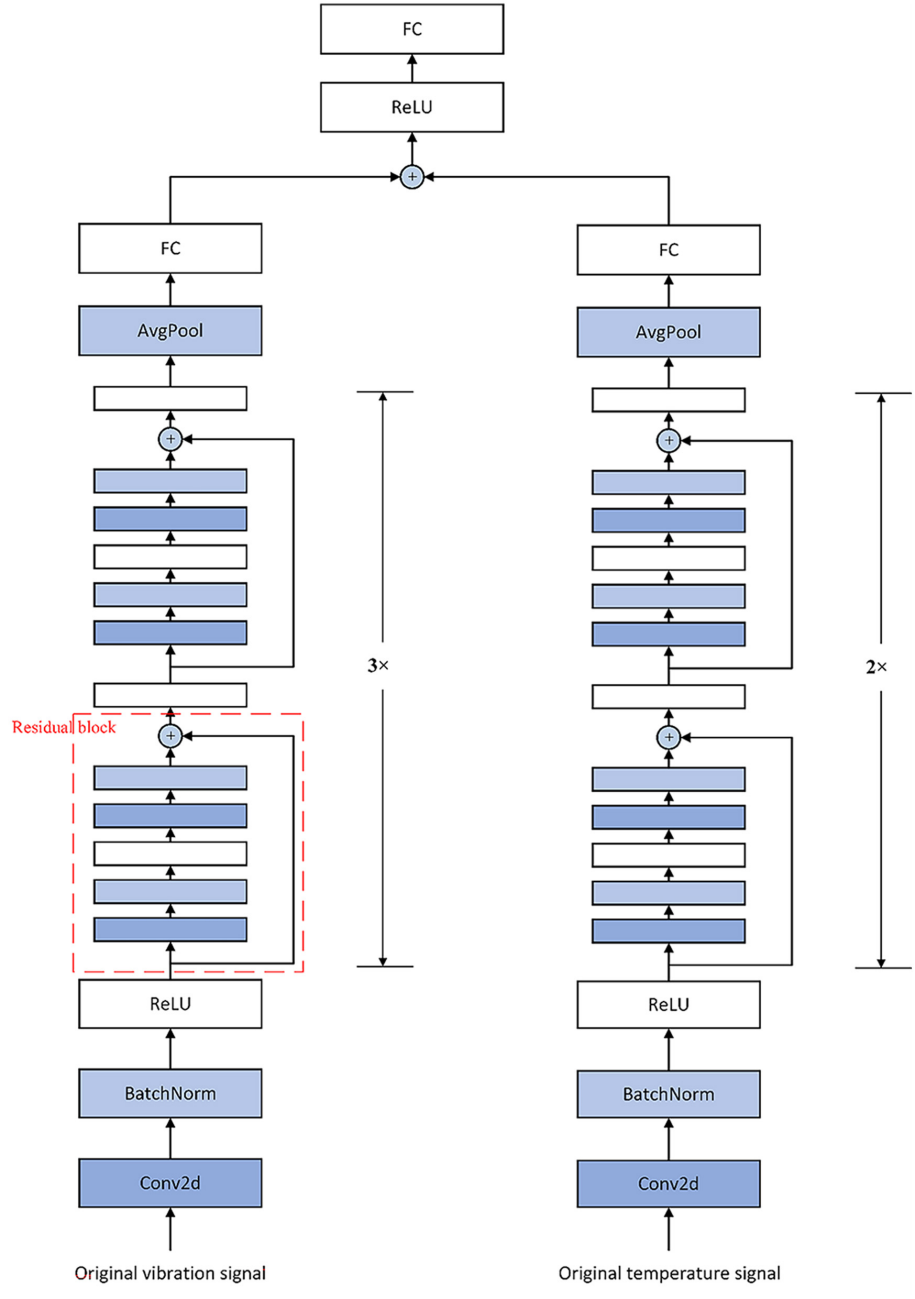
Specific structure of fusion model.

Because the signals collected in this study include vibration signal and temperature signal, and the sampling frequency and sampling point interval between the two signals are different, the decision-level fusion is selected for the fusion level when constructing the residual block of the residual neural network, so as to analyze and monitor the two groups of signals in real time and reduce the extra computing resources as much as possible. Finally, the specific model structure is shown in Figure 9.

Numerous hyperparameters and algorithmic components are fine-tuned throughout model architecture design and training to enhance predictive performance.

Batch normalization layers can not only accelerate the training process, but also reduce overfitting by adjusting the inputs for each layer to keep the mean stays close to 0 and the variance stays close to 1.

In addition, regularization can also control model complexity to prevent overfitting. Both L1 and L2 regularization operate by incorporating parameter magnitude (either absolute values or squared terms) into the loss function as penalty terms, effectively constraining model weights to control complexity. In this study, the L2 regularization method, also known as weight decay, was chosen, and the penalty term adopted in this method is the square of the parameter, which is able to make the weight of the model gradually approach 0, as shown in Equation 2.


$$\begin{array}{l} J\left(\theta \right)=L\left(\theta \right)+\alpha \overset{n}{\underset{i=1}{\sum }}{w_{i}}^{2} \end{array}$$ (2)

Where, *J*(θ) is the loss function with regularization, *L*(θ) is the original loss function, α is the regularization intensity, and *w*_*i*_ is the weight of the model.

The loss function plays a critical role in neural networks by measuring the discrepancy between model predictions and actual target values. Selecting an appropriate loss function significantly enhances both training effectiveness and model performance. For classification problems, cross-entropy loss is widely adopted due to its effectiveness in quantifying inter-class prediction errors and facilitating efficient parameter optimization. The cross-entropy loss is defined as follows:


$$\begin{array}{l} L\left(y,p\right)=-\sum_{i=1}^{k}y_{i}\log\left(p_{i}\right) \end{array}$$ (3)

Where, *L*(*y, p*) represents the cross-entropy loss, and *p*_*i*_ is the probability that the model prediction output belongs to category i.

The evaluation metrics of the regression model are different from those of classification tasks. Regression analysis deals with continuous numerical predictions, and traditional accuracy metrics are not applicable in this case. The standard evaluation criteria for regression performance include mean squared error (MSE), mean absolute error (MAE), and coefficient of determination (R^2^). In this study, MAE is adopted as the main loss function, which calculates the arithmetic mean of the absolute differences between the predicted values and the actual values. Its mathematical formula is as follows:


$$\begin{array}{l} \text{MAE}=\frac{1}{n}\overset{n}{\underset{i=1}{\sum }}|y_{i}-{ŷ}_{i}| \end{array}$$ (4)

The choice of optimization method significantly impacts parameter adjustment efficiency and convergence speed during model training. Among various optimizers like stochastic gradient descent (SGD), RMSprop, and AdaGrad, this research employs the Adam algorithm. Adam integrates momentum-based gradient descent with RMSprop's adaptive learning rate approach, dynamically modifying individual parameter learning rates through first-order (momentum term) and second-order (RMSprop term) gradient moment estimations.

The parameter update method of Adam algorithm is as follows (Kingma and Ba, 2014; Zaheer et al., 2018):

(1) Calculate the first moment estimation *m*_*t*_ and second moment estimation (RMSprop term) *v*_*t*_ corresponding to gradient *g*_*t*_.

$$\begin{array}{l} m_{t}=\beta_{1}\cdot m_{t-1}+\left(1-\beta_{1}\right)\cdot g_{t} \end{array}$$ (5)

$$\begin{array}{l} v_{t}=\beta_{2}\cdot v_{t-1}+\left(1-\beta_{2}\right)\cdot g_{t}^{2} \end{array}$$ (6)

(2) Correct the deviation between the first and second moment estimates:

$$\begin{array}{l} {\hat{m}}_{t}=\frac{m_{t}}{1-\beta_{1}^{t}} \end{array}$$ (7)

$$\begin{array}{l} {\hat{v}}_{t}=\frac{v_{t}}{1-\beta_{2}^{t}} \end{array}$$ (8)

(3) Update parameters

$$\begin{array}{l} \theta_{t+1}=\theta_{t}-\frac{\eta }{\sqrt{{\hat{v}}_{t}}+\varepsilon }\cdot {\hat{m}}_{t} \end{array}$$ (9)

Where, θ is the model parameter, η is the learning rate, and ϵ is a constant that usually takes the value of 1*e*−8.

## 4 Results and discussion

### 4.1 Model results

Model optimization is achieved through iterative training. The learning process essentially minimizes the objective function through an iterative process. Training begins with parameter initialization, followed by alternating forward and backward calculation cycles. In the forward calculation process, the input data propagates through the network layers to generate prediction results. Subsequently, the backward calculation computes the gradients of the loss with respect to all parameters, enabling the adjustment of weights, gradually reducing errors and improving model performance. The complete training algorithm is as follows.

(1) Initialize weights and biases. The weights and biases of nodes in the model need to be initialized before the training begins.
(2) Forward propagation. Forward calculations are performed based on the model structure and parameters to obtain the output of the model.

$$\begin{array}{l} a_{l}=g\left(\omega_{l}a_{l-1}+b_{l}\right) \end{array}$$ (10)

Where, *a*_*l*_ denotes the output of the *l*_th_ layer, ω_*l*_ refers to the weight of the *l*_th_ layer, *b*_*l*_ refers to the bias of the *l*_th_ layer, and *g*(·) is the activation function. The common activation functions are Tanh, Sigmod, and ReLu functions.

(3) Calculate the loss. The loss function is calculated based on the forward calculation result of the model and the actual label.

$$\begin{array}{l} J=\frac{1}{m}\sum_{i=1}^{m}L\left(y^{\left(i\right)},{ŷ}^{\left(i\right)}\right) \end{array}$$ (11)

Where, *m* represents the number of samples, *L*(·) refers to the loss function, *y*^(*i*)^ and ŷ^(*i*)^ represents the actual label and predicted result of the *i*_th_ sample, respectively.

(4) Backward propagation. The gradient of the model parameters can be calculated from the loss function through the following formulas.

$$\begin{array}{l} \frac{\partial J}{\partial \omega_{l}}=\frac{1}{m}\sum_{i=1}^{m}\frac{\partial L\left(y^{\left(i\right)},{ŷ}^{\left(i\right)}\right)}{\partial \omega_{l}} \end{array}$$ (12)

$$\begin{array}{l} \frac{\partial J}{\partial b_{l}}=\frac{1}{m}\sum_{i=1}^{m}\frac{\partial L\left(y^{\left(i\right)},{ŷ}^{\left(i\right)}\right)}{\partial b_{l}} \end{array}$$ (13)

(5) Parameter update. The optimization algorithm is adopted to update the model parameters, so that the parameters are optimized along the direction of decreasing the loss function.

During model training, steps (2)–(5) are executed cyclically until either loss convergence or predefined termination conditions are satisfied. This iterative optimization process systematically improves the model's ability to learn from training data.

This study evaluated various neural network architectures, with their respective accuracies presented in Table 5. As can be seen from Table 5, when the fused signal is the dataset, the classification accuracy of ResNet is the highest. When the single-source signal is the dataset, the classification accuracy of the same model with the fused signal is slightly higher. It can be concluded that the input of the fused signal into the ResNet model is the optimal model for this study. The comparative analysis reveals that the ResNet-based fusion model achieved superior performance, reaching a peak classification accuracy of 92.16%.

**Table 5:** Training results of different model structures.

**Model**	**Signal**	**Accuracy**
RBF-SVM	Fusion signal	82.44%
Random Forest	Fusion signal	85.04%
2DCNN	Fusion signal	89.36%
1DCNN+LSTM	Fusion signal	91.12%
ResNet	Temperature signal	78.24%
	Vibration signal	84.61%
	Fusion signal	92.16%

**Figure 10:**
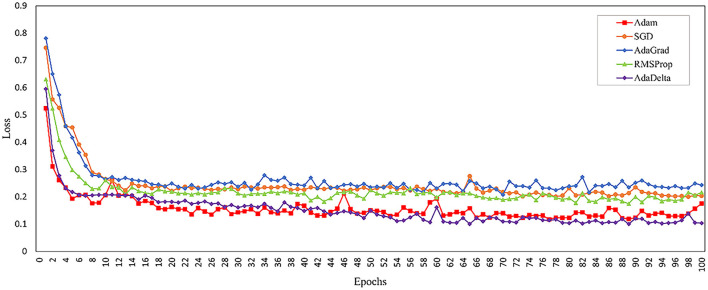
Graph of training loss for each optimizer.

Model optimization effectiveness and computational efficiency are highly dependent on the selected training algorithm. To select the most appropriate optimizer, this study compared the performance of different optimizers during the training of the same model for 100 epochs, as shown in Figure 10.

Comparative results in Table 6 demonstrate that the Adam optimizer achieves superior performance in both accuracy and computational efficiency for the ResNet fusion model among various optimization algorithms tested.

**Table 6:** The accuracy and training time of each optimization algorithm.

**Optimize algorithm**	**Accuracy**	**Training time**
Adam	92.16%	22,858.4 s
AdaGrad	90.27%	29,545.1 s
AdaDelta	91.45%	23,678.9 s
SGD	90.42%	32,541.8 s
RMSProp	91.22%	27,154.2 s

Through validation set evaluation, the fusion model's optimal architecture and parameters were determined, with results presented in Table 7.

**Table 7:** The fusion model structure and parameters.

**Model**		**Value**
Structure	Conv2d	1 × 800 × 86/1 × 54 × 43
	Residual block1	16 × 800 × 86/16 × 54 × 43
	Residual block2	32 × 400 × 43/32 × 27 × 21
	Residual block3	64 × 200 × 22/64 × 13 × 10
	AvgPool2d	64 × 200 × 22/64 × 13 × 10
	FC	64
	Classification FC	2
	Regression FC	5
Parameter	lr	0.001
	Epoch	100
	Batchsize	64
	Dropout	0.5

**Figure 11:**
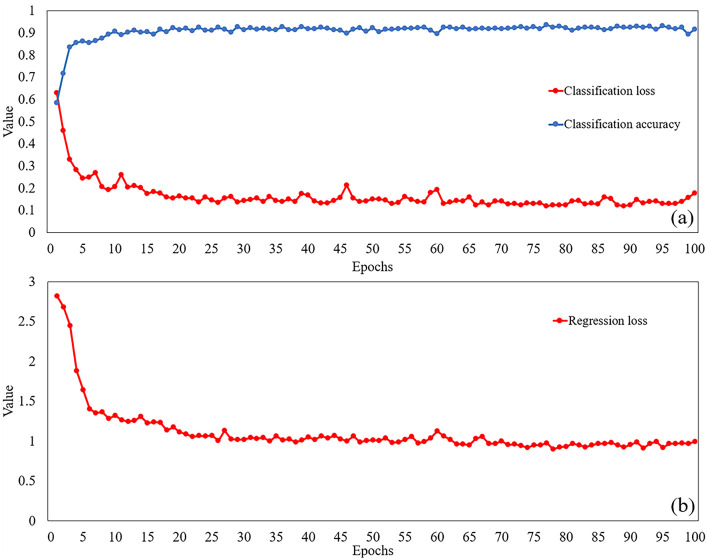
Classification **(a)** and regression **(b)** results of the model.

The performance of the optimized model is illustrated in Figure 11a, which displays both classification accuracy and loss for leak type identification, while Figure 11b depicts the regression loss results. In the classification task, as the number of training rounds increases, the classification loss drops rapidly, then fluctuates slightly at a lower level, the prediction error keeps decreasing, and eventually stabilizes, indicating that the model gradually converges in the classification task. The classification accuracy rose rapidly in the early stage of training and then gradually stabilized. As the number of training rounds increases, the regression loss drops rapidly and then fluctuates within a lower range. This indicates that the model's prediction accuracy in the regression task keeps improving, gradually optimizing from the initial large deviation, and stabilizing later, suggesting that the model also converges in the regression task.

The regression loss stabilizes near 1 after sufficient training iterations. Given that MAE measures the absolute difference between predictions and true values, this result suggests a mean positioning error of 1 meter, demonstrating the model's capability for meter-level leak detection. Implementing a helical fiber arrangement could enhance this accuracy even more.

We conducted a statistical test on the classification accuracy using the McNemar test. We sorted MAE results from 5-fold cross-validation and took the 2.5% and 97.5% percentiles as the 95% confidence interval. The specific results are as follows.


$$\begin{array}{l} x^{2}=\frac{{\left(b-c\right)}^{2}}{b+c}=0.0037 \end{array}$$ (14)

Based on the experimental data and through the McNemar test, the *p-value* is less than 0.05, indicating a significant difference between the two models. The MAE confidence interval of Resnet is [0.9107, 0.9526], while that of 1DCNN + LSTM is [1.0024, 1.1195]. Since the intervals do not overlap, it can be directly concluded that Resnet has better performance.

### 4.2 Dual-signal feature extraction and fusion (Findings for RQ2)

The single temperature signal has low sensitivity to minor leaks and is easily affected by environmental temperature variations, leading to incorrect judgments; the single vibration signal is susceptible to pipeline operation noise and difficult to distinguish between leakage vibrations and normal operating vibrations. Moreover, both single signal methods cannot fully extract the spatiotemporal correlation features of the leakage event, resulting in low accuracy of leakage identification. As RO2 pointed out, the core of integrating temperature signals with vibration signals lies in making these signals complement each other and work together to optimize. Through feature collaboration, leakage conditions can be identified, errors in individual signals can be avoided, and the integrity of feature extraction can be fundamentally improved.

**Figure 12:**
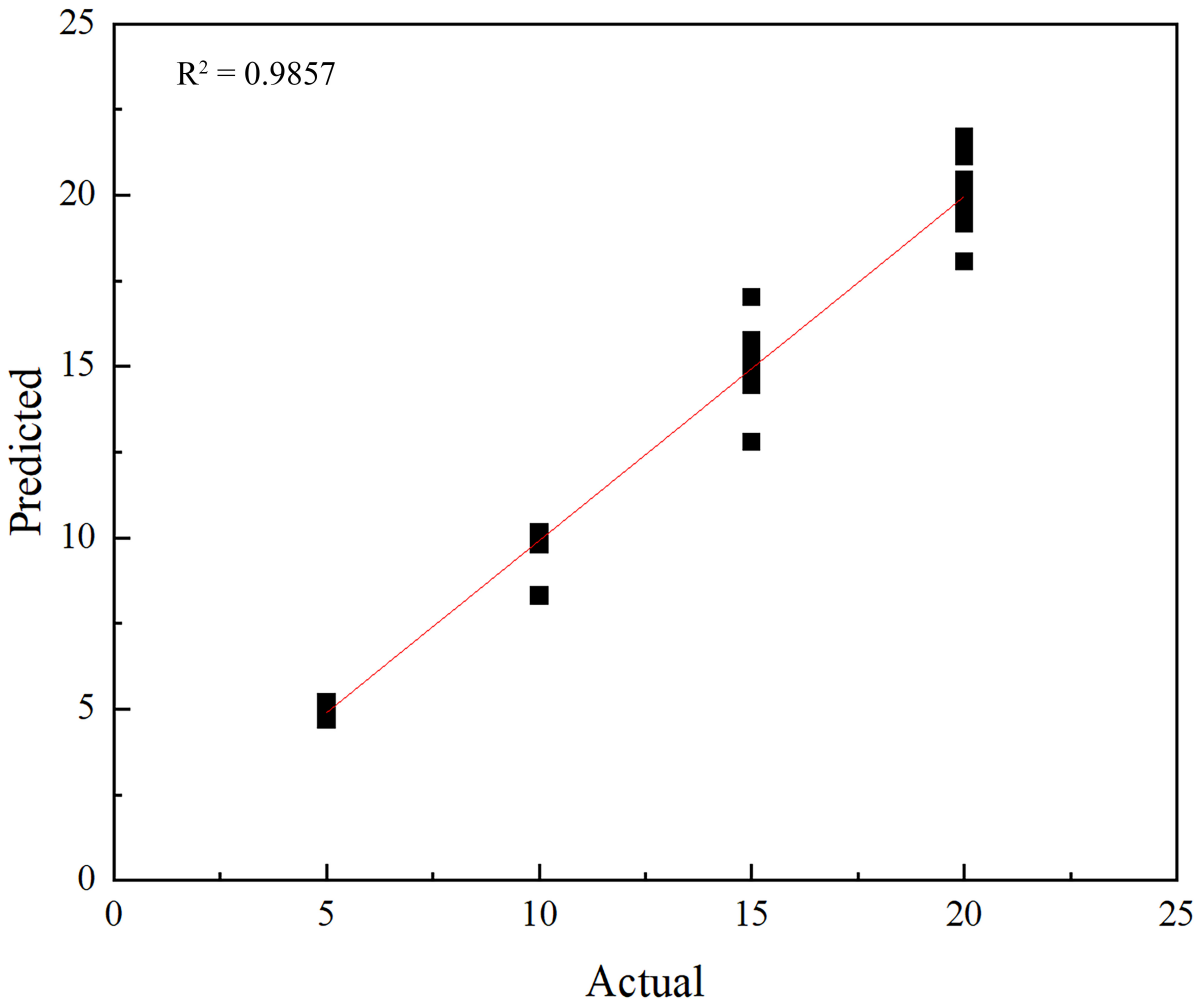
The positioning accuracy of the model.

In this study, residual networks were used to extract features from the original vibration signals and temperature signals separately, effectively learning and extracting the important features of the signals. The output of the residual network can yield two types of features: classification features and regression features. The classification features are used to classify the input signals, that is, to determine whether the signal belongs to the leakage category; while the regression features are used to locate the abnormal leakage points in the input signal, that is, to find the location where the leakage occurs. Through the decision-level fusion of the model, the classification features and regression features are concatenated to form a fused feature vector. The feature vector is classified through the fully connected layer to determine the state category of the signal. The fused feature vector is regressed through the linear layer to predict the final regression value, which is used for signal positioning. Through this deep learning model, this study can achieve intelligent state classification and positioning of the input signals without manual feature selection. However, the regression task usually does not use accuracy as an evaluation metric because the regression problem is the prediction of continuous numerical values and there is no concept of “correct” or “wrong” prediction. Therefore, the metric used to evaluate the performance of the regression model in this study is the mean absolute error. The output result of the regression task is the distance from the leakage location to the starting point of the pipeline. Figure 12 is the fitting graph for pipeline leakage location, which enables a clear visualization of the data distribution and the deviation between the model prediction and the actual values. In this study, the R^2^ value is 0.9857, indicating that the model positioning accuracy is quite good.

### 4.3 Potential applications

The above model provides a foundation for the construction of the monitoring system, enabling real-time monitoring and early warning of pipeline leaks. Moreover, by further integrating blockchain technology (Ressi et al., 2024), it can achieve automated alerts and maintenance workflows, avoiding the aggravation of pipeline corrosion, medium loss and environmental pollution caused by leakage, extending the effective lifespan of the pipeline, and also predicting potential pipeline failures (Doshmanziari et al., 2020), achieving the upgrade from “repair after failure” to “prevention before failure.” Through the closed loop of “physical entity—digital model—data interaction—decision feedback,” the full state digital mapping of the physical pipeline is realized (Grieves, 2005; Zhou et al., 2024; Huang et al., 2023).

## 5 Conclusions and prospect (Findings for RQ3)

In this study, temperature and vibration signals of distributed fiber optic systems under normal and leakage conditions were collected by the pipeline leakage experiment platform. In order to obtain more characteristic information about the pipeline operation time, as RO3 stated, this study proposes a residual network structure that integrates two parameters. This structure can simultaneously input the corresponding original optical fiber temperature and vibration signals. The two-dimensional convolution layer in the network model can extract and identify the temporal and spatial features of the signal, reduce manual participation, and realize the intelligent leakage monitoring of the oil pipeline. The training result shows that the ResNet model based on Adam optimization algorithm can achieve 92.16% leak identification accuracy, and the leak location accuracy reaches 1 meter. Based on this model, the comprehensive and real-time leakage identification and early warning of oil pipelines can be realized timely and accurately.

During our data collection process, due to the limitations of the experimental platform, it was not possible to simulate more complex service environments. In the future, we will further enrich our dataset in terms of pipeline length, pipe diameter, environmental interference, and the comparison of signals in straight and curved pipes. The research on vibration wave feature recognition methods will further expand the application boundaries and improve the structure and processing logic of the model, thereby enhancing the accuracy and reliability of the leakage assessment mechanism. In addition, because the viscosity of crude oil is much higher than that of water, it may cause significant differences in the flow velocity distribution and leakage volume within the pipeline. The experiment collected data without considering soil and weather conditions. The high moisture content of clay would enhance the attenuation of electromagnetic signals, resulting in errors in pipeline leakage location. Heavy rain weather would cause a sudden increase in soil moisture content, leading to baseline drift of the sensors. Low temperatures would freeze the outer walls of the pipelines, affecting the accuracy of temperature sensor data. The existing model training data covers a relatively short pipeline length, and an increase in pipeline length may lead to signal transmission attenuation. Verifying the generalization performance of the model in different scenarios of fluids, soil, weather, and pipeline lengths is necessary. Determining the applicable boundaries of the model can provide a basis for subsequent optimization. The specific verification plan can be found in Appendix A.

In the next 5 to 10 years, these three elements will form a deep synergy: Large-scale deployment will rely on IoT/micro-edge ubiquitous connections to achieve full-scenario coverage. The environmental robustness will break through the limitations of scenarios through transfer learning and edge adaptive algorithms, ultimately building an intelligent monitoring system of “deployment as a service, perception as decision-making, and environment as adaptation,” and achieving a paradigm shift from “passive response” to “active warning” in fields such as energy and municipal services (see Tables B1–B3). Furthermore, in order to trade-offs in model complexity and real-time application, we can structurally prune and quantize the model to make it more lightweight, reducing computational costs while maintaining accuracy. For embedded hardware commonly used in industry, the convolution operations in the residual network can be converted into pipelined calculations that can be processed in parallel by FPGA, or the CUDA cores of GPU can be utilized to accelerate spatiotemporal feature fusion. This enables the model to improve inference speed on low-cost hardware and increase the speed of real-time response for different types of events in low-load scenarios.

## Data Availability

The original contributions presented in the study are included in the article/supplementary material, further inquiries can be directed to the corresponding author.

## Appendix A. On-site verification plan

**Table A1:** Verification object and scenario design.

**Verification dimension**	**Scene type**	**Quantity/Scale**
Fluid type	Water, crude oil	Two verification pipelines each
Soil conditions	Clay (with moisture content of 25%) and sandy soil	Each consisting of 3 verification points
Weather conditions	Sunny weather, light rain (< 20mm/h), heavy rain (> 50mm/h)	Each lasts for 3 days of verification
Pipeline length	1 kilometers (short), 2 kilometers (medium), 3 kilometers (long)	One verification pipeline each

## Appendix B. Structured outlook (deployment, IoT/edge, environmental robustness)

**Table B1:** Stage-based deployment path.

**Stage**	**Objective**	**Key task**	**Time**
Pilot verification	Verify the feasibility of the core scenario	Select 3 to 5 typical scenarios (oil pipeline + sandy soil), and complete the model and hardware compatibility testing	1 to 2 years
Regional promotion	Formulate a standardized deployment plan	Establish hardware installation guidelines and model parameter adjustment manuals, and replicate them in the same type of areas	2 to 3 years
Cross-domain scalability	Covering multiple scenarios and the entire chain link	Integrate the transfer learning module to achieve rapid deployment across scenarios for fluids, soils, and lengths, and form an industry solution.	3 to 5 years

**Table B2:** Internet of things/evolution path of edge technologies.

**Level**	**Current status**	**Mid-term goals (3–5 years)**	**Long-term goals (5–10 years)**
Edge node	With a single function (such as data collection)	Integrated with lightweight AI chips, supporting local inference	Possessing self-repairing capability (automatically switching to the backup module in case of hardware failure)
Internet of things network	Wireless as a supplement to wired (LoRa)	5G slice dedicated network coverage, transmission delay ≤ 50ms	6G ubiquitous connectivity, supporting ultra-long-range edge collaboration up to 100 kilometers
Cloud-edge collaboration	Periodic model update (monthly)	Real-time Federated Learning (Hourly Parameter Synchronization)	Self-evolutionary collaboration (where edge nodes autonomously initiate model optimization requests)

**Table B3:** Prospects for environmental resilience.

**Environmental dimension**	**Current challenges**	**Breakthrough technology**	**Expected outcome**
Extreme climate	Heavy rain or intense heat exposure	For the pipe optical fibers, a double-layer sheath structure is adopted, and a real-time signal calibration algorithm is employed.	The optical fiber signal attenuation is controlled within 0.2 dB/km, and the failure rate is reduced to below 0.1% per year.
Complex geology	The signal attenuation in the clay zone leads to inaccurate positioning.	Adaptive signal enhancement algorithm (adjusts the sensor's transmission power dynamically according to soil resistivity)	Reduction of positioning error
Fluid diversity	Interference of crude oil impurities on model predictions	Fluid feature alignment module based on transfer learning (real-time correction of density / viscosity to mitigate the impact on the model)	The accuracy rate of cross-fluid type prediction remains above 90%.
